# Cognitive impairments in Moroccan man with a frontal anaplasic oligodendroglioma : Case study

**DOI:** 10.1192/j.eurpsy.2023.2031

**Published:** 2023-07-19

**Authors:** M. Legmouz, F. Z. Azzaoui, A. El Ouahabi

**Affiliations:** 1 Biology and health, Ibn tofail University, Kenitra; 2 Neurosurgery, Hopital des spécialitéss, Rabat, Morocco

## Abstract

**Introduction:**

Oligodendroglioma is a rare form of glioma developing in oligodendrocytes which are glial cells. Oligodendrogliomas are divided into two types; benign oligodendrogliomas and anaplastic oligodendrogliomas; the malignant form.

**Objectives:**

The aim of our study is to detect the cognitive troubles caused by a frontal anaplasic oligodendroglioma in a man admitted to the neurosurgery department at the Specialties Hospital, Rabat, Morocco.

**Methods:**

A case study was realized among a man aged of 46 years , suffering from frontal right anaplasic oligodendroglioma, with no medical and surgical history and who presented since two months headaches and forgetfulness, and presented two hours ago a fortuitous epileptic seizure followed by notion of amnesia for 5 min. At the admission, the patient was conscious, the glasgow score was of 15 and did not present a motor deficit. Cognitively, the patient was confused. We decided then to make him pass the MOCA test to evaluate his cognitive state as soon as he was admitted to our service and before the surgical act .

**Results:**

After passing the MOCA Test, our patient had a score of 4/30. The results in detail gave 0/5 in the visuospatial/executive part, 3/3 in the naming part, 0/6 in the attention part, 1/3 in the language part, 0/2 in the abstraction part, 0/5 in the memory part and 0/6 in the orientation part. The score of 4/30 is less than 10/30 and shows a severe cognitive impairment.

According to his wife, his cognitive state was normal before the epileptic seizure and had a normal life.

**Conclusions:**

The frontal anaplastic oligodendroglioma in this patient case deteriorated his cognitive state rapidly. 13 days after surgery and excision of this tumor, the patient’s cognitive state improved, the Moca score became 12/30, which is in the area of moderate cognitive impairment. The patient is currently continuing his treatment in an oncology department.

**Disclosure of Interest:**

None Declared

